# Oral microbiome alterations in epilepsy and after seizure control

**DOI:** 10.3389/fmicb.2023.1277022

**Published:** 2023-11-29

**Authors:** Xiaolei Lian, Zhenguo Liu, Tianwen Wu, Jiamin Lou, Yuan Chen, Shanshuo Liu, Limin Jin, Shuang Li, Yajun Lian, Yan Jiang, Zhigang Ren

**Affiliations:** ^1^Department of Neurology, The First Affiliated Hospital of Zhengzhou University, Zhengzhou, China; ^2^The Academy of Medical Sciences of Zhengzhou University, Zhengzhou University, Zhengzhou, China; ^3^State Key Laboratory of Organ Failure Research, Guangdong Provincial Key Laboratory of Viral Hepatitis Research, Department of Infectious Diseases, Nanfang Hospital, Southern Medical University, Guangzhou, China; ^4^Department of Infectious Diseases, The First Affiliated Hospital of Zhengzhou University, Zhengzhou, China

**Keywords:** epilepsy, seizure control, oral microbiome, diagnostic model, receiver operating characteristic curve

## Abstract

**Background:**

The existing diagnostic methods of epilepsy such as history collection and electroencephalogram have great limitations in practice, so more reliable and less difficult diagnostic methods are needed.

**Methods:**

By characterizing oral microbiota in patients diagnosed with epilepsy (EPs) and patients whose seizures were under control (EPRs), we sought to discover biomarkers for different disease states. 16S rRNA gene sequencing was performed on 480 tongue swabs [157 EPs, 22 EPRs, and 301 healthy controls (HCs)].

**Results:**

Compared with normal individuals, patients with epilepsy exhibit increased alpha diversity in their oral microbiota, and the oral microbial communities of the two groups demonstrate significant beta diversity differences. EPs exhibit a significant increase in the abundance of 26 genera, including *Streptococcus*, *Granulicatella*, and *Kluyvera*, while the abundance of 14 genera, including *Peptostreptococcus*, *Neisseria*, and *Schaalia*, is significantly reduced. The area under the receiver operating characteristic curve (AUC) of oral microbial markers in the training cohort and validation cohort was 98.85% and 97.23%, respectively. Importantly, the AUC of the biomarker set achieved 92.44% of additional independent validation sets. In addition, EPRs also have their own unique oral community.

**Conclusion:**

This study describes the characterization of the oral microbiome in EP and EPR and demonstrates the potential of the specific microbiome as a non-invasive diagnostic tool for epilepsy.

## 1 Introduction

Epilepsy is a chronic, non-communicable disease that affects people of all ages, with approximately 5 million people globally diagnosed with this neurological disorder each year (World Health Organization, 2019). There is no single, simple, and reliable diagnostic method for epilepsy, and a complete history and reliable eyewitness accounts are key to diagnosis (Thijs et al., 2019). In addition, polymorphisms in epilepsy-related genes and social and economic factors complicate diagnosis. The interval from the first seizure to a well-defined seizure can range from weeks to decades (Jallon et al., 2001; Firkin et al., 2015). Unfortunately, delayed treatment and misdiagnosis of epilepsy are not uncommon and potentially devastating (Firkin et al., 2015; Xu et al., 2016).

Gut microbes and the brain communicate with each other via vagus nerve activation, endocrine and immune response regulation, and microbial metabolite production (Amlerova et al., 2021). Neurological and psychiatric disorders occur when changes such as genetic polymorphisms, environmental stimuli, dietary changes, and gastrointestinal disorders affect the brain-gut axis (Walker et al., 2020). For example, animal studies by Olson et al. (2018) clearly showed that specific intestinal flora mediates the antiepileptic effects of a ketogenic diet. Several human studies have shown that the composition of the intestinal microbiota in people with refractory epilepsy is altered compared with that of healthy people (Peng et al., 2018; Gong et al., 2020; Holmes et al., 2020).

The oral microbiome is a microbiome library that continuously supplements the gut microbiome, and at least one-third of oral microbiome cells enter the intestinal tract of healthy people through the digestive tract (Iwauchi et al., 2019; Schmidt et al., 2019). A 16S rRNA gene sequencing study based on 27 children with cerebral palsy and epilepsy showed a clear correlation between intestinal and oral microbiota (Huang et al., 2022). This reflects the importance of oral microbiota in epilepsy. In addition, oral microorganisms and their products may also enter the brain through the systemic circulation or peripheral nerves (Horowitz et al., 2004; Kook et al., 2014). However, the characteristics of the oral microbiota in patients with epilepsy remain unclear.

Based on the results of previous intestinal microbiota, we attempted to further reveal changes in the oral microbiota in epilepsy patients using a large cohort and identify related biomarkers. We hope that the exploration of oral microbiota will provide new ideas for the diagnosis and treatment of epilepsy.

## 2 Experimental section

### 2.1 Research program

This study was approved by the Institutional Review Board of the First Affiliated Hospital of Zhengzhou University (No. 2021-KY-0574-002). All samples and clinical data involved in this study were collected with the informed consent from each participant. From early 2019 to late 2020, 161 tongue swab samples were collected from 161 hospitalized patients diagnosed with epilepsy (EPs). In total, 161 EPs were all newly diagnosed with epilepsy by a neurologist. Out of the 161 patients with epilepsy, a subset of 22 was followed up for 3–4 months, and during this follow-up, we observed that these 22 patients achieved seizure control through drug treatment. At the conclusion of the follow-up period, tongue swabs were collected from these 22 patients whose seizures were under control (EPRs). In the second half of 2021, we collected corresponding tongue swabs from 29 patients with newly diagnosed epilepsy recruited from the same urban district as an independent test cohort. EP and EPR were selected by neurology professionals concerning our inclusion and exclusion criteria. The healthy controls (HCs) consisted of 527 volunteers who underwent a physical examination at the First Affiliated Hospital of Zhengzhou University from 2019 to 2020. The inclusion and exclusion criteria of subjects are presented in Supplementary methods. The tongue swabs were analyzed using 16S rRNA gene sequencing on the MiSeq platform.

### 2.2 Tongue swab collection

As described by Lu et al. (2022), a sterile swab was used to obtain the tongue flora. Routine examinations (including oral examinations) were performed on the first day of admission, and tongue swabs were collected the next morning before brushing teeth and eating breakfast. Before tongue swab samples are taken, a general oral examination is performed by the dental hygienist in the physical examination department through probing, percussion, palpation, and looseness. Its content includes (World Health Organization, 2019) observing the integrity and symmetry of the maxillofacial region and whether there are developmental malformations (Thijs et al., 2019); checking all teeth for caries and defects (Firkin et al., 2015); checking the oral mucosa and tongue for ulcer, cracked tongue, and lichen planus (Jallon et al., 2001); checking the growth of impacted teeth and whether it harms adjacent teeth or other tissues (Xu et al., 2016); checking whether there are lumps in the oral cavity and whether there are abnormal conditions such as leukoplakia. Samples will be discarded if the patient has periodontitis, dental caries, oral ulcer, and other oral diseases. After each subject was gargled, the operator used a one-time throat swab to scrape from the back to the front middle area of their tongue coating. After the operation, the throat swab was put into a sterile cryotube and then immediately stored in the refrigerator at −80°C.

### 2.3 DNA extraction and polymerase chain reaction amplification

As usual, we extracted DNA from the microbes using the Qiagen Mini Kit (Qiagen, Hilden, Germany) following the manufacturer’s instructions (Ren et al., 2014). The DNA concentration was measured by NanoDrop (Thermo Scientific), and the molecular size was estimated by agarose gel electrophoresis. 301F (5′-CCTACGGGNGGCWGCAG-3′) and 805R (5′-GACTACHVGGGTATCTAATCC-3′) primers were used for polymerase chain reaction (PCR) amplification of the V3–V4 region of the extracted 16S rRNA gene. The amplification process in the ABI GeneAmp^®^ 9700 PCR thermocycler (ABI, CA, United States) is as follows: initial denaturation was performed at 95°C for 3 min; the process involved denaturation at 95°C for 30 s, annealing at 55°C for 30 s, and extension at 72°C for 45 s, which were repeated for 27 cycles; followed by a single extension at 72°C for 10 min. Finally, the reaction was kept at 4°C. The reaction system consisted of template DNA (10 ng), TransStart FastPfu DNA Polymerase (0.4 μL), 5 μM forward primer (0.8 μL), 5 μM reverse primer (0.8 μL), 5 × TransStart FastPfu buffer (4 μL), 2.5 mM dNTPs (2 μL), and ddH_2_O (up to 20 μL). PCR products were detected using 2% W/V agarose gel, and bands were extracted and purified by AxyPrep DNA Gel (Axygen, CA, United States) and the PCR Clean-Up System.

### 2.4 MiSeq sequencing and sequence data processing

The DNA library was built according to the manufacturer’s instructions. The gene sequences were sequenced on an Illumina MiSeq platform of Shanghai Mobio Biomedical Technology Co., Ltd., China. The amplicon reading was processed by following these steps: (a) the double-ended sequencing readings for each library are overlapped using FLASH version 1.2.10, with default parameters (Magoč and Salzberg, 2011); (b) The following rules are defined to make FLASH-generated overlapping reads perform stricter quality control: (1) ambiguous bases (N) were not allowed in the reading process; (2) five or more mismatches were prohibited in the overlapping region; (3) mismatches in the barcode primer region were not allowed; (c) the reads were demultiplexed and then assigned to different samples with barcodes; (d) after discovering and removing chimeric sequences using UCHIME 4.2.40 (Edgar et al., 2011), the Human Oral Microbiome Database^1^ was used as a reference for matching operational taxonomic units (OTUs).

### 2.5 OTU clustering and taxonomy annotation

An equal number of reads were randomly selected from each sample, and then OTUs were binned through the UPARSE pipeline. We ensure that OTUs include all samples collected at different times. The similarity threshold is set to 97%. Finally, according to the developer’s documentation, we use the RDP classifier V.2.6 to annotate the sequences.

### 2.6 Bacterial diversity and taxonomic analysis

The alpha diversity and beta diversity of microorganisms were obtained based on the OTU analysis of samples. The alpha diversity including the Ace index, Chao index, Shannon index, Simpson index and observed OTUs were calculated by the R program package “vegan.” Non-metric multidimensional scaling (NMDS), principal component analysis (PCA), and principal coordinate analysis (PCoA) were analyzed by R packet^2^ to show differences in the microbiome between different groups. A heatmap showing the key OTU between the two groups is generated by the heat map builder.

The Wilcoxon rank-sum test was used to compare the two groups of bacteria in different phylum and genus categories. Linear discriminant analysis (LDA) effect size (LEfSe)^3^ first uses the Kruskal–Wallis rank-sum test (*p* < 0.05) to find statistically significant variables and then evaluates the magnitude of the impact of each variable on the differential effect between the two groups by LDA [LDA score (log10) >3]. For *p*-values, multiple testing was performed using the false discovery rate.

### 2.7 Functional annotation of 16S rRNA gene-based on the Kyoto encyclopedia of genes and genomes profile

The KEGG orthology (KO) and KEGG pathway/module maps were built using PICRUSt2^4^ and Human version 0.99 (Abubucker et al., 2012). Then, combined with the 16S rRNA gene sequence of the sample, we predicted the functional profiles of the microbial community.

### 2.8 Construction and validation of the diagnostic model

OTU frequency profiles for different queues are generated by mapping the readings from the corresponding queues to these representative sequences. In the discovery cohort, the Wilcoxon rank-sum test was first used to determine the significance (*p* < 0.05), to obtain OTU with inter-group differences. Based on the difference in the OTU abundance profile of the discovery queue, 5-fold cross-validation is carried out on the random forest model, where all parameters are default values, except for importance = TRUE. The determined set of optimal OTUs is used to calculate the probability of the disease index of the discovery queue and the validation queue. The R package (R 3.3.0, pROC package) was used to construct the receiver operating characteristic (ROC) curve to evaluate the diagnostic capability of the constructed model, where the area under the receiver operating characteristic curve (AUC) represents the ROC effect (see Supplementary methods for the detailed model building process).

### 2.9 Statistical analysis

For the basic information of the participants, the following statistical analysis methods were used. The continuous and categorical variables were expressed as mean (standard deviation) and percentage, respectively. Tongue swab samples from EPs (*n* = 100) and HCs (*n* = 200) were compared. For the two groups of continuous variables with independent design, the two-sample *t*-test was applied for comparison between the groups assuming normal distribution and homogeneity of variance, and the approximate *t*-test was used for comparison between the groups assuming normal distribution and non-homogeneity of variance. The statistical analysis was performed using SPSS V.26 for Windows (SPSS, Chicago, Illinois, United States). Statistical significance was defined by *p* < 0.05 (two-tailed), without post-analysis and alpha adjustment.

## 3 Results

### 3.1 The research protocol and flow chart

**Figure 1:**
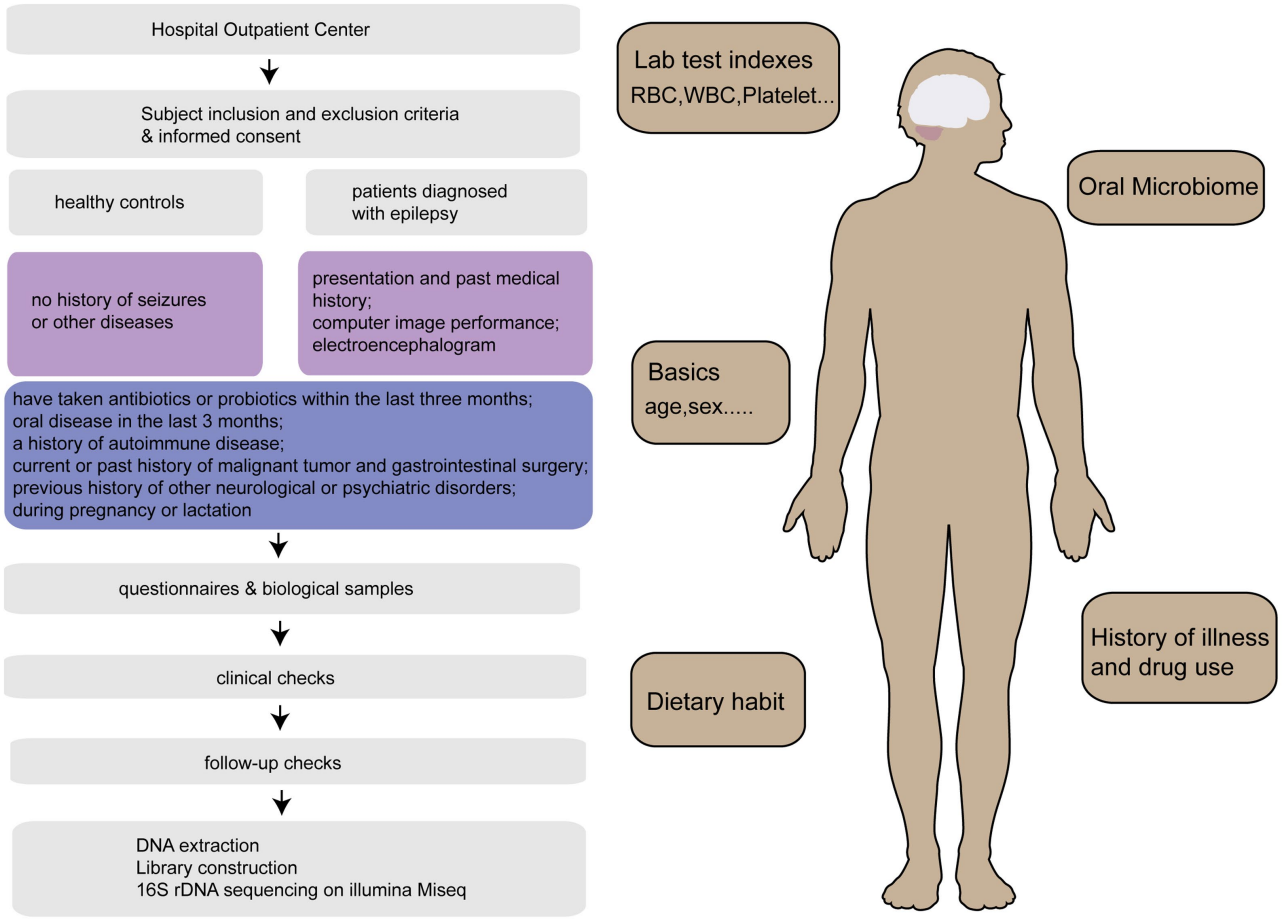
Research program. After strict inclusion and exclusion of 515 tongue swabs collected from Henan Province, 509 tongue swabs [157 patients diagnosed with epilepsy (EPs), 22 patients whose seizures were under control (EPRs), 301 healthy controls (HCs), 29 EPs] were analyzed. HCs, healthy controls; EPs, patients diagnosed with epilepsy; EPRs, patients whose seizures were under control; RFC, random forest classifier.

A total of 480 samples [157 patients diagnosed with epilepsy (EPs), 22 patients whose seizures were under control (EPRs), and 301 healthy controls (HCs)] from Henan Province were included in this study for statistical analysis. We randomly selected tongue swab samples at the ratio of 2:1 as the discovery cohort and verification cohort. The specific research framework is shown in Figure 1. First, we characterized the oral microbiomes of 100 EPs and 200 HCs as a discovery cohort, identified different microbes and their key metabolic pathways between the two groups, and obtained the best biomarkers using random forest and 5-fold cross-validation. Furthermore, we verified the diagnostic capability of the classifier with tongue swabs of 57 EPs and 101 HCs. It is worth noting that 29 EPs included at different time points were taken as independent test queues to further verify the reliability of the diagnostic model. We also explored the oral microecology of 22 EPRs and 44 HCs (20 EPs and 19 EPRs).

### 3.2 Basic information and clinical characteristics of participants

The basic information of 100 EPs and 200 HCs for oral samples is shown in Supplementary Table S1. There was no significant difference in sex or age between EP and HC (*p* < 0.05). EP exhibited statistically significant differences in red blood cell (RBC) count, white blood cell (WBC) count, hemoglobin, albumin, uric acid (UA), estimated glomerular filtration rate (eGFR), and total bilirubin (TBIL) compared with HC (*p* > 0.05), but these values were all within the normal range.

### 3.3 Oral microbiological characteristics of EP and HC

**Figure 2:**
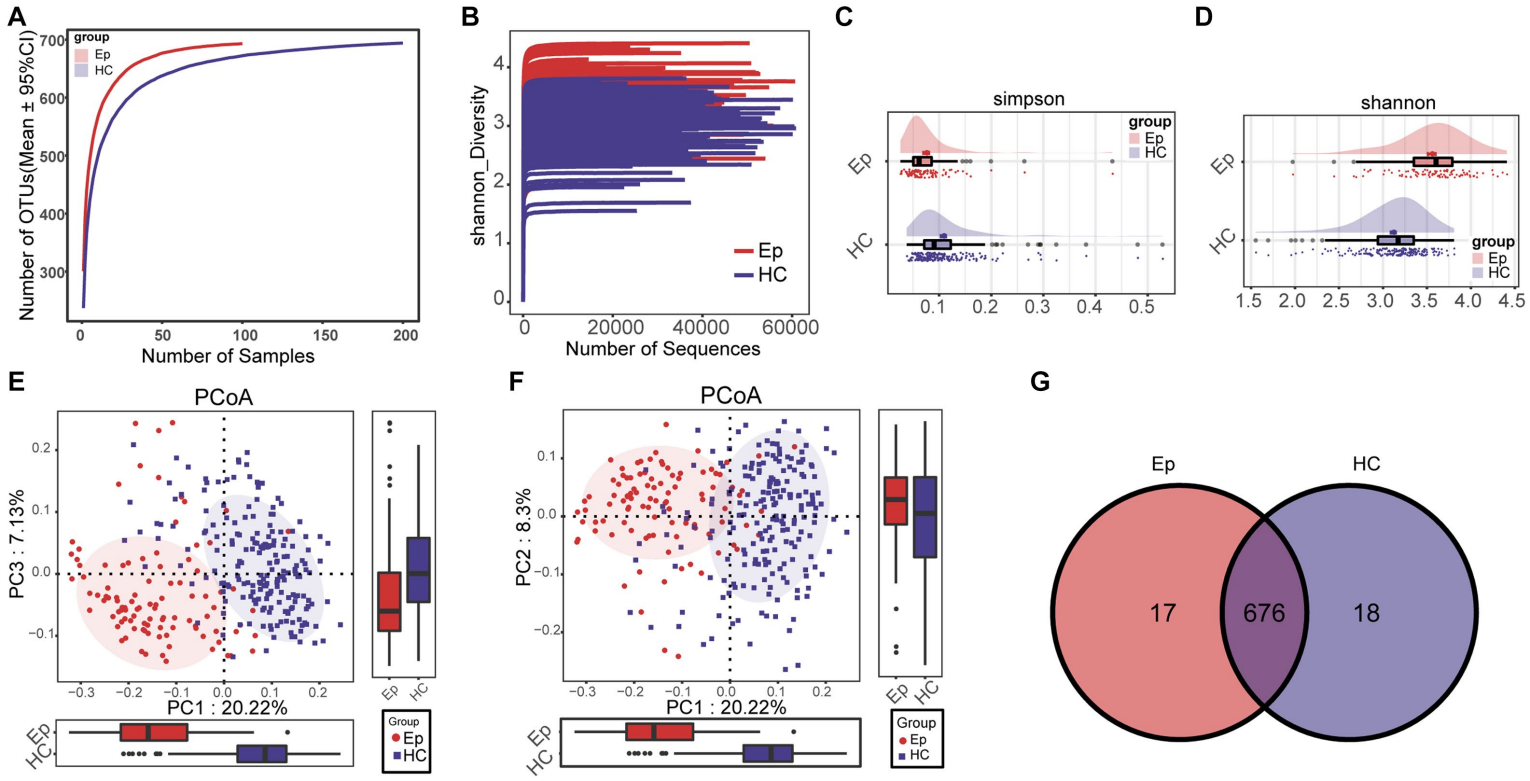
Differences between alpha diversity and beta diversity of oral microorganisms between EP and HC. **(A)** The species accumulation curve showed that the number of OTUs remained at a constant level with increasing sample size, i.e., the sample size was sufficient. **(B)** Shannon–Wiener indicated that with the increase in sequencing amount, the microbial diversity index of each sample did not increase significantly, that is, the sequencing amount was sufficient. **(C)** The Simpson index showed that EP had a higher diversity of colonies than HC. **(D)** The Shannon index showed that EP (*n* = 100) had higher microbial diversity than HC (*n* = 200). **(E)** PCoA (PC1–PC3) analysis using unweighted UniFrac showed significant differences in community structure between EP and HC. **(F)** PCoA (PC1–PC2) showed differences in community structure between EP and HC. **(G)** The Venn diagram shows the number of OTUs shared by EP and HC and the number of OTUs unique to each. OTUs, operational taxonomic units; PCoA, principal coordinate analysis; HC, healthy control; EPs, patients diagnosed with epilepsy.

In the oral microecological discovery cohort (100 EPs and 200 HCs), the species accumulation curve and Shannon–Wiener curve showed that the sample size and sequencing data were sufficient for both groups (Figures 2A,B). Regarding alpha diversity, the Shannon index and Simpson index indicated that compared with HC, the abundance and uniformity of oral microbes were increased in EP (Figures 2C,D; Supplementary Table S2). Beta diversity was employed to compare the microbial community composition. Principal Coordinate Analysis (PCoA) was conducted based on the distance matrix calculated by unweighted UniFrac. The analysis revealed significant microbial community separation between the two groups (Figures 2E,F). According to a Venn diagram, 676 operational taxonomic units (OTUs) were shared by EP and HC, with 17 OTUs in EP and 18 OTUs in HC (Figure 2G).

**Figure 3:**
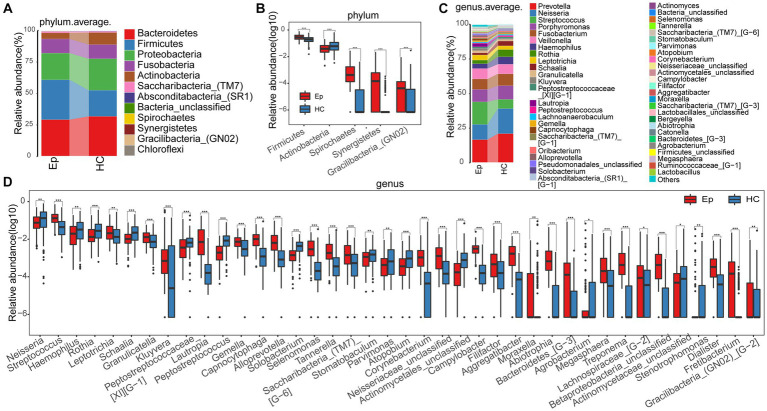
Composition and differences of oral microbes in EP and HC at the phylum and genus levels, respectively. **(A)** At the phylum level, EP and HC mean species composition distributions. **(B)** At the phylum level, there were statistically significant microorganisms in the relative abundance between EP and HC. **(C)** At the genus level, the average species composition of EP and HC was distributed. **(D)** At the genus level, there was a significant difference between EP and HC in oral microorganisms. EPs, patients diagnosed with epilepsy; HC, healthy control.

At the phylum level, the mean species composition distribution of different groups and the bacterial communities with significant differences between groups are shown in Figure 3A (Supplementary Table S5) and Figure 3B (Supplementary Table S6). In EP, the relative abundance of *Firmicutes*, *Spirochetes*, and another two phyla increased, while the relative abundance of *Actinobacteria* decreased. At the genus level (Figure 3C), the average species composition of *Prevotella*, *Neisseria*, *Streptococcus*, *Porphyromonas*, *Fusobacterium*, and *Veillonella* in EP and HC was 68.044% and 71.193%, respectively (Supplementary Table S3). Compared with HC, the relative degrees of 26 genera, including *Lautropia*, *Capnocytophaga*, and *Selenomonas,* were significantly increased in EP. However, the relative abundance of *Peptostreptococcus*, *Solobacterium*, *Atopobium*, and the other 11 genera decreased significantly (Figure 3D; Supplementary Table S4).

**Figure 4:**
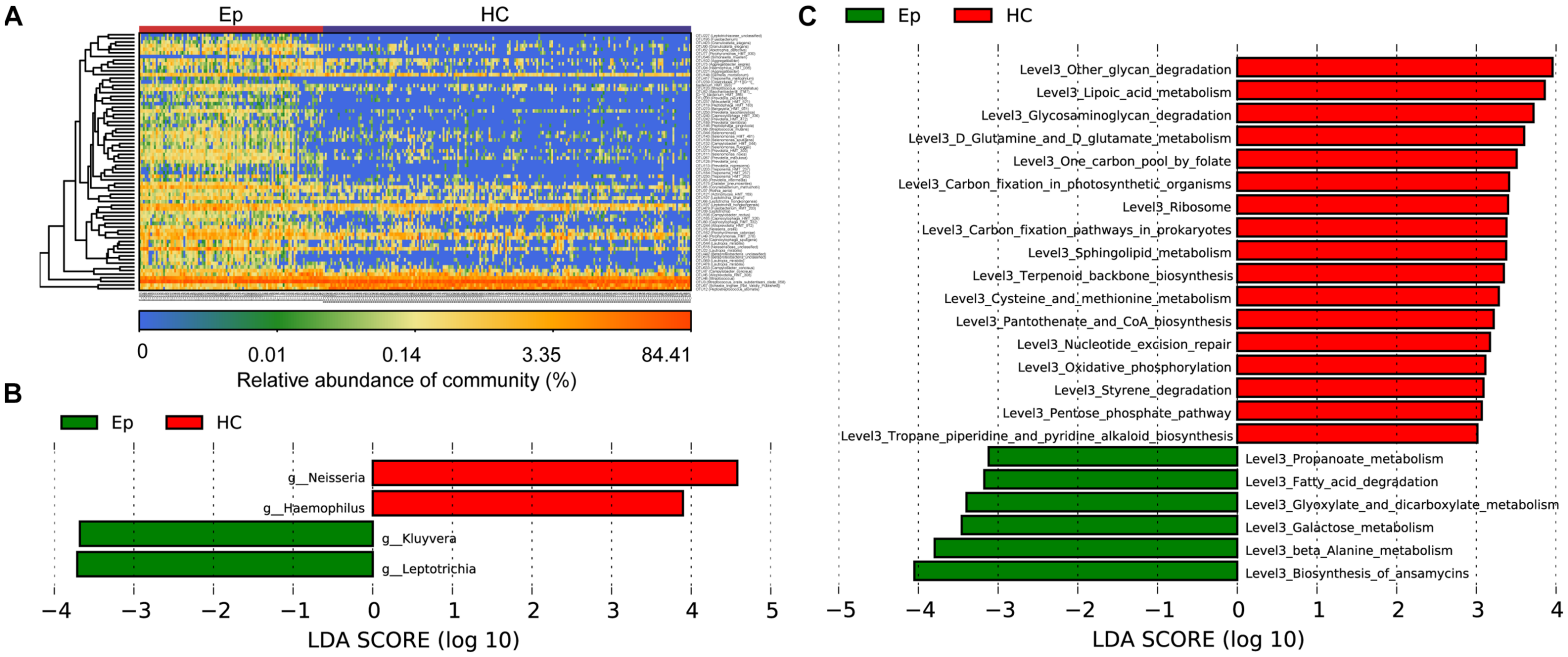
Key oral microbes and microbial functions associated with EP. **(A)** The heat map shows the relative abundance of different microorganisms in each sample. **(B)** The histogram of LDA value distribution shows the microorganisms with a significant difference at the genus level between the two groups (*p* < 0.05, LDA >3). **(C)** Histogram of LDA value distribution shows significant differences in microbial function at the L3 level between the two groups (*p* < 0.05, LDA >3). EPs, patients diagnosed with epilepsy; HC, healthy control; LDA, linear discriminant analysis.

A heatmap (Figure 4A; Supplementary Table S7) was drawn based on the abundance of information on OTUs in each sample with significant differences between the two groups. We observed enrichment of 68 OTUs in EP and 4 OTUs in HC. Linear discriminant analysis (LDA) effect size (LEfSe), based on the effect size of LDA, was employed to further screen out microorganisms (*p* < 0.05, LDA >3) (Figure 4B; Supplementary Table S8). Additionally, the Kyoto Encyclopedia of Genes and Genomes (KEGG) metabolic pathway (*p* < 0.05, LDA > 3) (Figure 4C) most related to the difference between groups was identified. In EP, biosynthesis of ansamycins, galactose metabolism, and 4 other metabolic pathways was enriched; 17 metabolic pathways, such as lipoic acid metabolism and other glycan degradation, were enriched in HC (Supplementary Table S9).

### 3.4 Diagnosis model of epilepsy based on the oral microbiome

**Figure 5:**
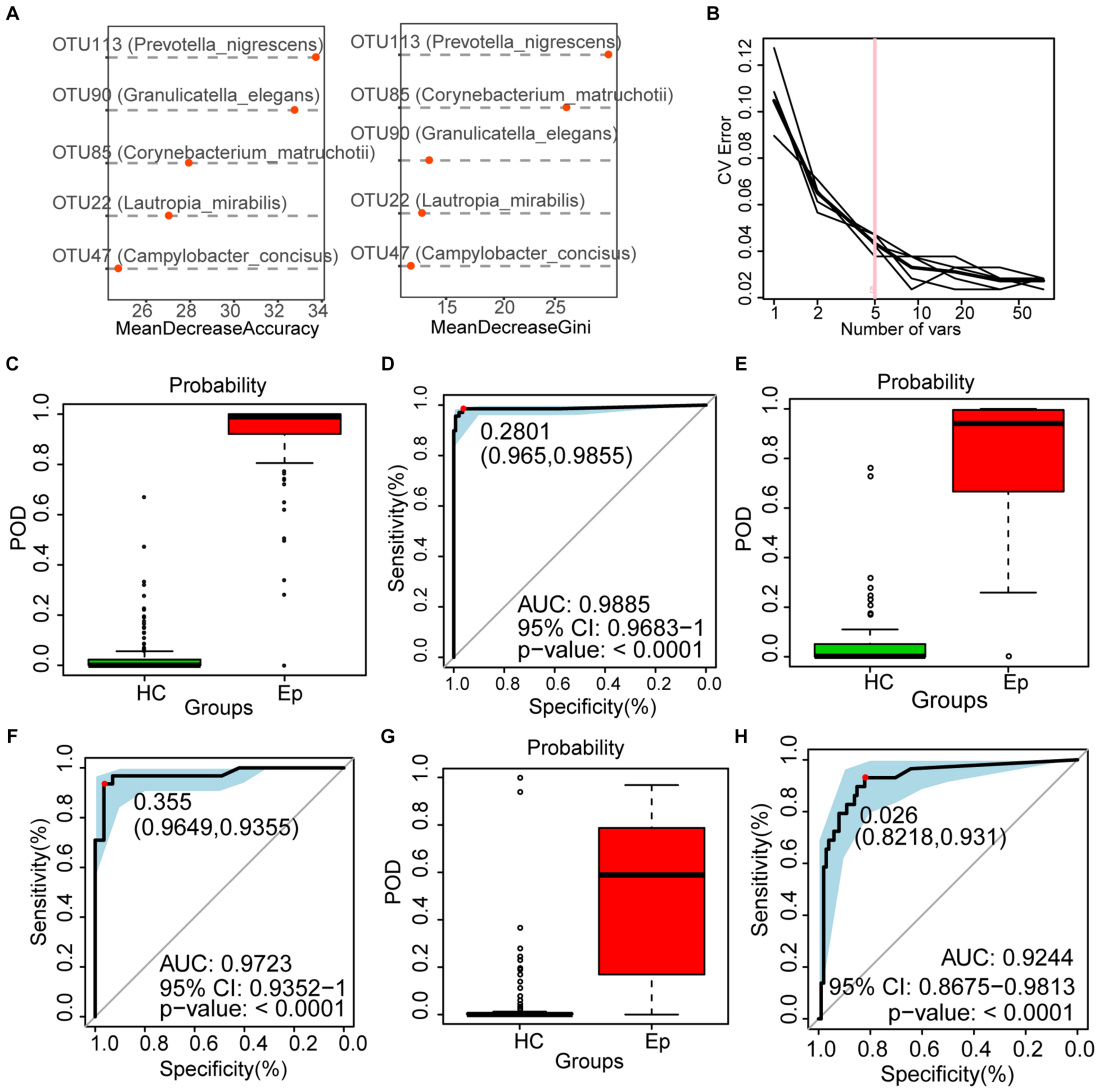
To construct a diagnosis model of epilepsy based on the oral microecology. **(A,B)** Five OTUs were selected as the best biomarkers. **(C)** The POD value of EP was significantly higher than that of HC in the discovery cohort. **(D)** In the discovery cohort, the AUC was 98.85%. **(E)** In the validation cohort, the POD value of EP (*n* = 57) was significantly higher than that of HC (*n* = 101). **(F)** In the validation cohort, the AUC was 97.23%. **(G)** In the independent test cohort, the POD value of EP (*n* = 29) was significantly higher than that of HC (*n* = 101). **(H)** In the validation cohort, the AUC was 92.44%. OTUs, operational taxonomic units; POD, probability of disease; AUC, area under the ROC curve; HC, healthy control; EPs, patients diagnosed with epilepsy.

The random forest analysis was performed on the discovery cohort to build a diagnostic model, which was further corrected by 5-fold cross-validation to obtain the most accurate OTU combination (Figures 5A,B). Based on the best biomarker comprising five OTUs, the probability of disease (POD) index of EP was significantly higher than that of HC (Figure 5C); the area under the receiver operating characteristic curve (AUC) reached 98.85% (95% CI: 96.83 to 100%, *p* < 0.0001) (Figure 5D). Furthermore, 57 EPs and 101 HCs in the validation cohort supported the diagnostic value of the best tag set. In the validation cohort, the POD index of EP was higher than that of HC (Figure 5E), and the AUC reached 97.23% (95% CI: 93.52 to 100%, *p* < 0.0001) (Figure 5F). To further confirm the validity of the diagnostic model, 29 EPs from Zhengzhou (Henan Province) were included at different time points as an independent test cohort. The POD index of EP was higher than that of HC (Figure 5G), and the AUC reached 92.44% (95% CI: 86.75% to 98.13%, *p* < 0.0001) (Figure 5H). These results suggest that oral microbiology-based markers can be used as a non-invasive tool for the diagnosis of epilepsy.

### 3.5 Oral microbiological characteristics of EPR and HC

**Figure 6:**
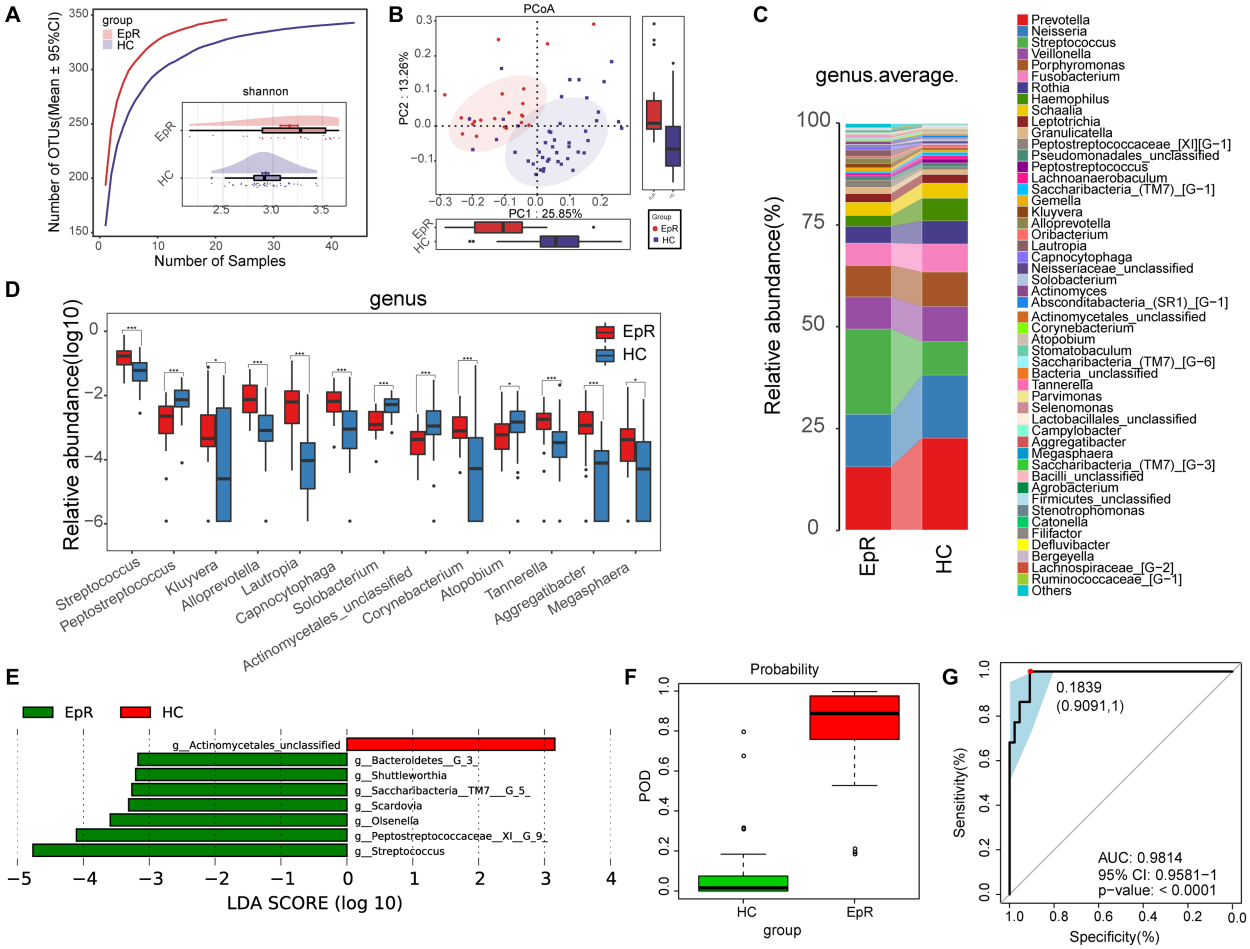
Microecological characteristics of EPR patients are different from those of HCs. **(A)** The species accumulation curve showed that the number of OTUs did not increase significantly with increasing sample size. The Shannon index indicated that the microbial diversity of EPR (*n* = 22) was higher than that of HC (*n* = 44). **(B)** PCoA analysis using unweighted UniFrac showed differences in the community structure between EPR patients and HCs. **(C)** The mean species composition distribution of EPR and HC at the genus level. **(D)** Microorganisms with significant differences in the relative abundance between EPR and HC at the genus level. **(E)** Histogram of LDA value distribution shows microorganisms closely related to the two groups at the genus level (*p* < 0.05, LDA >3). **(F)** In the discovery cohort, the POD value of EPR is higher than that of HC. **(G)** In the discovery cohort, the AUC was 98.14% ^*^p < 0.05, ^**^p < 0.01, and ^***^p < 0.001. OTUs, operational taxonomic units; PCoA, principal coordinate analysis; LDA, linear discriminant analysis; POD, probability of disease; AUC, area under the ROC curve; HC, healthy control; EPRs, patients whose seizures were under control. Centerline, median; box limits, upper and lower quartiles; circle or square symbol, mean; error bars, 95% CI.

We followed previously enrolled EP and eventually collected tongue swabs from 22 EPRs. Under the premise of sufficient sample size and sequencing data for both groups (Figure 6A; Supplementary Figure S1A), alpha diversity suggested higher oral microbial diversity for EPR than HC (Figure 6A; Supplementary Figure S1B; Supplementary Table S10). PCoA showed that the two groups had different microbial communities (Figure 6B; Supplementary Figure S1C). As shown in the Venn diagram in Supplementary Figure S1D, EPR and HC had 327 OTUs together, with 19 OTUs and 16 OTUs, respectively.

The mean species composition distribution and bacteria with statistical significance at the genus (phylum) level between the two groups are shown in Figure 6C (Supplementary Table S11) and Figure 6D (Supplementary Table S12) [Supplementary Figure S1G (Supplementary Table S13) and Supplementary Figure S1H (Supplementary Table S14)]. Compared with HC, the relative abundance in EPR was increased for 8 genera and decreased for 5 genera (Figure 5D).

A heatmap (Supplementary Figure S1E; Supplementary Table S15) indicated that 49 OTUs were enriched in EPR and 4 OTUs were enriched in HC. LEfSe further screened out microorganisms closely related to intergroup differences (*p* < 0.05, LDA >3) (Figure 6E; Supplementary Table S16). Compared with HC, 9 metabolic pathways (e.g., bacterial chemotaxis, synthesis, and degradation of ketone bodies, and benzoate degradation) were active in EPR, while 12 metabolic pathways were less active (Supplementary Figure S1F; Supplementary Table S17).

Through a 5-fold cross-validation analysis of the key OTUs, we obtained the best tag set that could accurately distinguish the two groups (Supplementary Figures S1I,J). The POD index of EPR was significantly higher than that of HC (Figure 6F), and the AUC reached 98.14% (95% CI: 95.81% to 100%, *p* < 0.0001) (Figure 6G). Hence, the oral microflora of EPR patients differs from that of HC.

### 3.6 Oral microbial alterations before and after seizure control

**Figure 7:**
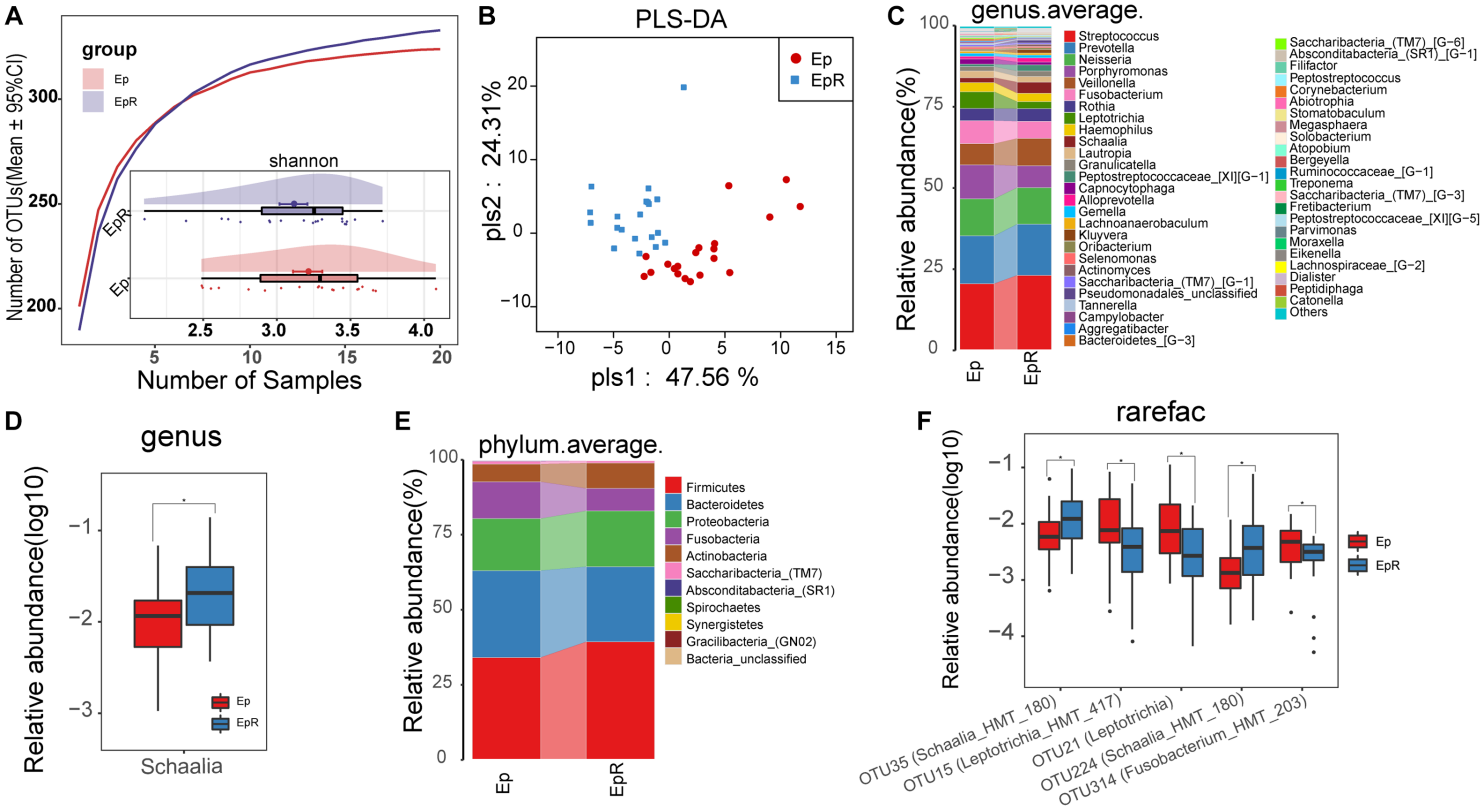
Microecological differences before and after seizure control. **(A)** The species accumulation curve showed that the number of OTUs did not increase significantly with increasing sample size. The Shannon index showed no significant difference in microbial diversity between EPR (*n* = 20) and EP (*n* = 20). **(B)** PLS-DA analysis showed differences in community structure between EP and EPR. **(C)** At the genus level, the average species composition of EP and EPR was distributed. **(D)** At the genus level, there were statistically significant microorganisms in the relative abundance between EP and EPR. **(E)** At the phylum level, EP and HC mean species composition distributions. **(F)** The box plot shows OTUs with a significant difference in the relative abundance between the two groups ^*^p < 0.05, ^**^p < 0.01, and ^***^p < 0.001. OTUs, operational taxonomic units; PLS-DA, partial least squares discriminant analysis; POD, probability of disease; AUC, area under the ROC curve; EPRs, patients whose seizures were under control. Centerline, median; box limits, upper and lower quartiles; circle or square symbol, mean; error bars, 95% CI.

To determine the changes in oral microflora before and after seizure control, we characterized the oral microecology of 20 EPs and 19 EPRs. Under the premise of sufficient sample size and sequencing volume (Figure 7A; Supplementary Figure S2A), there was no significant difference in microbial alpha diversity between EP and EPR (Figure 7A; Supplementary Figure S2B; Supplementary Table 18). As a supervised pattern recognition method, PLS-DA (Figure 7B) revealed differences in microbial community composition between the two groups. As shown in Supplementary Figure S2C, the Venn diagram shows 318 OTUs in both EP and EPR, 6 OTUs in EP, and 15 OTUs in EPR. Figures 7C,E (Supplementary Tables S19, S21) illustrate the average species composition of EP and EPR at the genus and phylum levels. Compared with EPR, the relative abundance of *Schaalia* in EP decreased (Figure 7D; Supplementary Table S20).

Among the differential OTUs screened by the Wilcoxon rank-sum test (Figure 7F; Supplementary Table S22), the relative abundance of three OTUs [e.g., OTU15 (*Leptotrichia_HMT_417*), OTU21 (*Leptotrichia*)] in EPR was lower than that of EP, but the relative abundance of OTU35 (*Schaalia_HMT_180*) and OTU224 (*Schaalia_HMT_180*) was higher.

## 4 Discussion

In this study, we compared the oral microflora of a large sample of patients with epilepsy to that of healthy subjects and identified differences in the oral microflora of the two groups. This study showed that the oral microbiome diversity of patients with epilepsy was higher than that of healthy controls, but there was no statistically significant difference in oral microbiome diversity before and after seizure control, even though the values were reduced. In the study by Gong et al. (2021), this is similar to the alpha diversity of intestinal microflora for children with refractory epilepsy treated with a ketogenic diet. However Peng et al. (2018), found no significant difference in alpha diversity of the intestinal microflora in drug-sensitive epilepsy compared with a healthy state.

Compared with HC, the relative abundance of *Streptococcus* and *Leptotrichia* in EP increased, while the relative abundance of *Neisseria*, *Haemophilus*, *Rothia*, and *Schaalia* decreased. However, the relative abundance of *Streptococcus* in EPR still increased after seizure control compared with HC. In a retrospective analysis of 145 children with central nervous system infections, the cerebrospinal fluid culture of 26.1% of infected patients showed the presence of *Group B Streptococcus* and *Streptococcus pneumoniae* (Lin et al., 2019). The growth of *Streptococcus* in the gut affects the levels of interleukin-6 and tumor necrosis factor-alpha (Jiang et al., 2015) and contributes to neurodegenerative diseases by inducing neuroinflammation (Huang et al., 2019). As normal oropharyngeal flora, commensal Neisseria may affect the colonization of potential pathogens (Dorey et al., 2019). The relative abundance of *Schaalia* in EPR returned to normal levels. *Schaalia* is one of the oral bacteria that produce nitrite (Sato-Suzuki et al., 2020), which has antibacterial activity and the ability to improve systemic blood circulation (Lundberg and Weitzberg, 2010). The relative abundance of *Kluyvera* and *Lautropia* in the EPR was not restored. *Kluyvera* is mainly colonized in the respiratory tract, urinary tract, and gastrointestinal tract (Braunstein et al., 1980). Although it has not been reported to infect the central nervous system, there have been some cases of blood infection (Linares et al., 2000). These microorganisms may be a preferred target for intervention therapy in epilepsy. We think that the return to normal levels of flora may play an important role in epileptic seizures. The presence of flora that remains at abnormal levels suggests that there may still be an inherent pathogenic biochemical basis for EPR.

Antiepileptic drugs can affect the composition of gut microbiota, thereby regulating the metabolism of central neurotransmitters (Lum et al., 2020; Li et al., 2022). The oral microbiome in this study changed after seizure control, which may be due to the direct effect of antiepileptic drugs. However, oral microbiota may also change after seizure control under the combined effects of reduced stress and immune system regulation, which needs to be further explored and confirmed by long-term longitudinal studies. Genomic technologies have significantly broadened our understanding of the role of oral microbes in different diseases (Sun et al., 2022). Specific sets of oral microbiotas have been shown to have strong diagnostic power in novel coronavirus (Ren et al., 2021), autoimmune hepatitis (Rao et al., 2021), rheumatoid arthritis (Zhang et al., 2015), systemic lupus erythematosus (Guo et al., 2023), cholangiocarcinoma (Rao et al., 2022), lung cancer (Sun et al., 2023), and colorectal cancer (Flemer et al., 2018). We used random forest analysis to construct relevant biomarkers based on oral microbes and validated the diagnostic efficacy of the model with a randomized cohort. This diagnostic model will be helpful for early screening or diagnosis of epilepsy, especially when the diagnosis of epilepsy is not clear, and it may be an important auxiliary means. Since we included epileptic seizure control subjects who were seizure-free in a short period of time, further study on patients who have recovered is needed.

This study has some limitations. Although cost-effective 16S rRNA gene sequencing is expected to increase the utility of disease diagnostic models, it cannot capture information about fungi and viruses, as well as metagenomics. In addition, the oral microbiota is susceptible to various factors; while this study validated the established biomarkers with an independent cohort recruited at different time points, additional external cohorts from diverse regions are necessary for future validation. The patients with epilepsy who were recruited in our study were those who had received an initial clinical diagnosis of epilepsy. However, due to the polymorphism and concealment of epilepsy, it is also possible that the disease state of epilepsy itself has existed for some time. In addition, the status of disease persistence reflects the progression of the disease, which may affect the study results. However, the large cohort and the clinical selection of patients with an initial diagnosis of epilepsy minimized this effect. In future, the effect of epilepsy duration on oral microbiota needs to be further explored.

In conclusion, based on tongue coat microflora analysis, this study identified changes in oral microflora among EP, EPR, and HC and constructed biomarkers for each condition. It is important to note that the specific oral microflora reflect the changes and characteristics of the disease in different states of epilepsy and seizure control.

## Data availability statement

The data presented in the study are deposited in the European Bioinformatics Institute European Nucleotide Archive database, accession number PRJNA759716.

## Ethics statement

The studies involving humans were approved by the Institutional Review Board of the First Affiliated Hospital of Zhengzhou University (No. 2021-KY-0574-002). The studies were conducted in accordance with the local legislation and institutional requirements. Written informed consent for participation in this study was provided by the participants’ legal guardians/next of kin.

## Author contributions

XL: Data curation, Formal analysis, Writing – review & editing. ZL: Data curation, Writing – original draft. TW: Resources, Software, Writing – review & editing. JL: Data curation, Resources, Writing – review & editing. YC: Methodology, Resources, Writing – review & editing. SsL: Resources, Writing – review & editing. LJ: Investigation, Resources, Writing – review & editing. ShL: Resources, Writing – review & editing. YL: Investigation, Methodology, Resources, Writing – review & editing. YJ: Investigation, Methodology, Resources, Writing – review & editing. ZR: Data curation, Funding acquisition, Writing – review & editing.

## Footnotes

^1^ www.homd.org

^2^ http://www.R-project.org/

^3^ http://huttenhower.sph.harvard.edu/lefse/

^4^ https://github.com/picrust/picrust2

## Glossary

EPs: Patients diagnosed with epilepsy
EPRs: Patients whose seizures were under control
HCs: Healthy controls
PDE-ALDH7A1: Pyridoxine-dependent epilepsy
AUC: Area under the receiver operating characteristic curve
OTU: Operational taxonomic unit
ROC: Receiver operating characteristic curve
RBC: Red blood cell
WBC: White blood cell
ALB: Albumin
BUN: Blood urea nitrogen
eGFR: Estimated glomerular filtration rate
T-chol: Total cholesterol
TG: Triglyceride
TBIL: Total bilirubin
PCoA: Principal coordinate analysis
LDA: Linear discriminant analysis
POD: Probability of disease
KEGG: Kyoto encyclopedia of genes and genomes
