# Supplementary material for: Oral microbiome alterations in epilepsy and after seizure control
Source: Front Microbiol. 2023 Nov 29;14:1277022. doi: 10.3389/fmicb.2023.1277022 (PMC10721976; doi:10.3389/fmicb.2023.1277022)
Supplement: Supplementary file 2 [file Data_Sheet_1.pdf]

Supplementary method

Subject inclusion and exclusion criteria

1. Inclusion criteria for patients with confirmed epilepsy(1)

(1) At least two unprovoked (or reflex) seizures occurring > 24 hours apart; (2) one unprovoked (or reflex) seizure and a probability of further seizures similar to the general recurrence risk (at least 60%) after two unprovoked seizures, occurring over the next 10 years; (3) diagnosis of an epilepsy syndrome.

2. Inclusion criteria for epileptic seizure control patients

Patients diagnosed with epilepsy are given medication on the recommendation of a professional and do not have seizures for three to four months.

3. Exclusion criteria for epileptic patients, seizure control patients, and healthy subjects

(1) Have received antibiotics, probiotics, prebiotics, or vitamin, protein, and unsaturated fatty acids within the last 3 months; (2) have suffered from oral diseases In recent 3 months; (3) A history of multiple sclerosis, neuromyelitis optica, systemic lupus erythematosus, rheumatoid arthritis, and other autoimmune diseases; (4) Subjects with a current or past history of malignancy and gastrointestinal surgery; (5) A history of neurological or psychiatric disorders such as Parkinson's disease, Alzheimer's disease, anxiety disorder, depression, autism spectrum disorder, schizophrenia; (6) There are

other metabolic diseases such as hypertension, diabetes, obesity, and metabolic syndrome; (7) Pregnant or breastfeeding; (8) Severe malnutrition, infection, drug or alcohol abuse.

Diagnostic model construction steps

1. Random Forest and OTU Importance Values: Initially, all OTUs were input into a Random Forest model to calculate the importance value (Mean Decrease Accuracy) for each OTU. These importance values reflect the contribution of each OTU to the classification model's performance, and the OTUs were sorted in descending order based on their importance values.
2. Gradually Increasing OTU Count and Calculating Error Rates: Starting from the sorted list of OTUs, the OTU count was incrementally increased, and diagnostic models were constructed using these OTUs. For each different number of OTUs, the error rate of the constructed diagnostic model was calculated. This provided insights into how the model's performance changed with the increasing number of OTUs
3. 5-Fold Cross-Validation and Error Rate Curves: 5-fold cross-validation was performed on the training set to obtain 5 different error rate curves for cross-validation. These curves demonstrated the performance of the models built using different numbers of OTUs across different cross-validation folds.
4. Calculating Average Error Rates and Cut-Off Value: Using the data from the 5 error rate curves, the average error rate of cross-validation was computed, and a curve representing the average values was plotted. The minimum error rate from the average

curve, plus the standard deviation of the cross-validation error rates, was selected as the cut-off value. This cut-off value served as a threshold for selecting the optimal set of OTU markers.

5. Selecting the Optimal Set of OTU Markers: Based on the cut-off value, all OTU marker sets with cross-validation error rates below the cut-off were listed. Then, the set with the fewest OTUs was chosen as the optimal set. This approach aimed to select a minimal number of OTUs while maintaining a low error rate for the optimal model.

References

1. Fisher RS, Acevedo C, Arzimanoglou A, Bogacz A, Cross JH, Elger CE, Engel J, Jr., Forsgren L, French JA, Glynn M, Hesdorffer DC, Lee BI, Mathern GW, Moshé SL, Perucca E, Scheffer IE, Tomson T, Watanabe M, Wiebe S. 2014. ILAE official report: a practical clinical definition of epilepsy. Epilepsia 55:475-82.

Supplementary Figure S1-S2

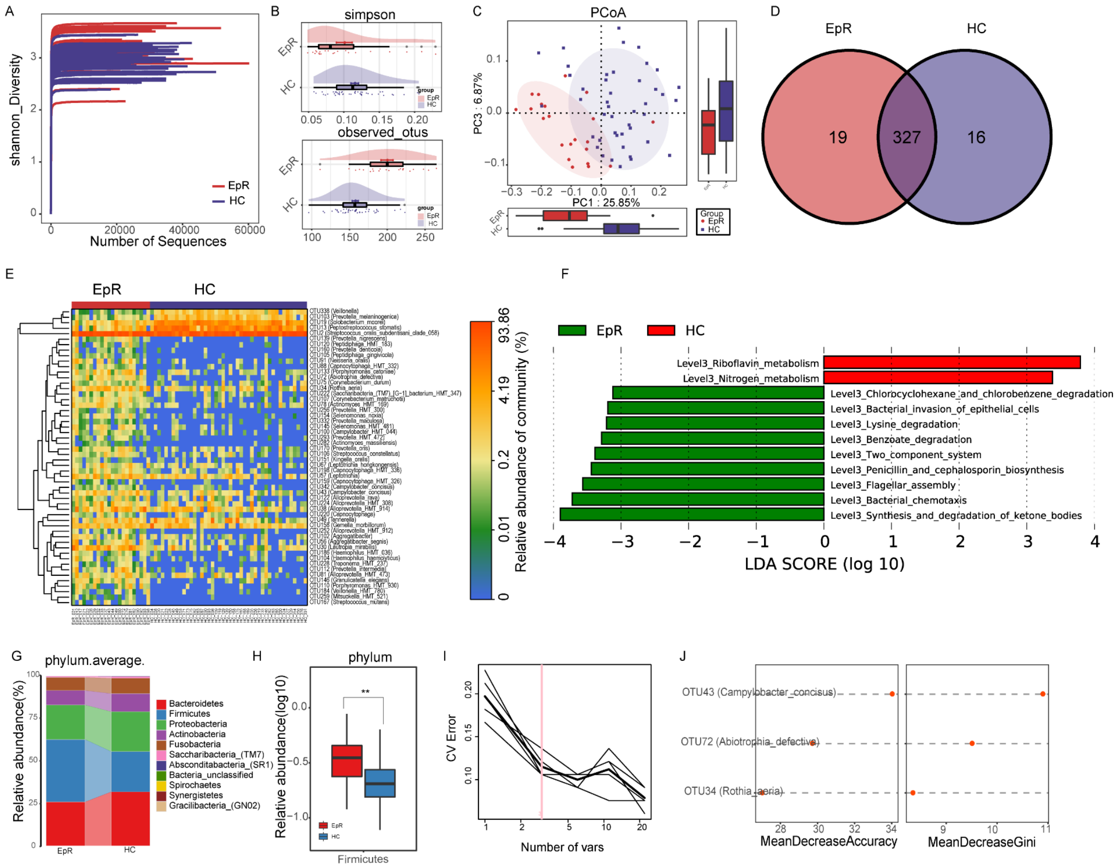

Figure S1. The microecological characteristics of EPR (n=22) are different from HC (n=44). (A) Shannon-wiener indicated that the microbial diversity index of the two groups remained constant with the increase of sequencing amount. (B) Simpson index showed that EPR had higher colony diversity than HC. Observed OTU showed that the species richness of EPR was higher than HC. (C) Venn diagram shows the number of OTUs shared by EPR and HC and the number of OTUs unique to each (D) PCoA showed significant differences in community structure between EPR and HC. (E) The heatmap shows the relative abundance of different microorganisms in each sample. (F) At the L3 level, the histogram of LDA value distribution shows microbial

functions closely related to the two groups ($p < 0.05$, LDA > 3). (G) At the phylum level, the mean species composition distribution of EPR and HC. (H) At the phylum level, there was statistically significant microorganism in relative abundance between EPR and HC. (I and J) Three OTUs were selected as the best biomarkers. *, $p < 0.05$, **, $p < 0.01$, ***, $p < 0.001$. OTUs, Operational Taxonomic Units; PCoA, Principal Co-ordinates Analysis; LDA, linear discriminant analysis; HC, healthy control; EPR, patient whose seizures are under control. Centerline, median; box limits, upper and lower quartiles; circle or square symbol, mean; error bars, 95% CI.

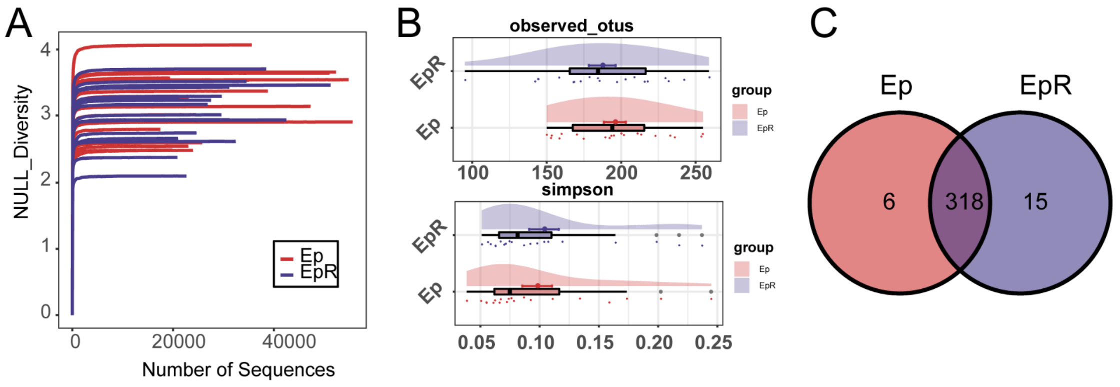

Figure S2. Microecological differences before and after seizure control (EPs, n=20; EPRs, n=20). (A) Shannon-wiener indicated that the microbial diversity index of each sample did not increase with the increase of sequencing amount. (B) Observed OTU showed that there was no statistical difference in species richness between EP and EPR. Simpson index showed that there was no statistical difference in colony diversity between EP and EPR. (C) The Venn diagram shows the number of OTUs shared by EP and EPR and the number of OTUs unique to each. OTUs, Operational Taxonomic Units; EP, patient diagnosed with epilepsy; EPR, patients whose seizures are under control. Centerline, median; box limits, upper and lower quartiles; circle or square symbol, mean; error bars, 95% CI.
